# Evolutionary history of genus *Coptis* and its dynamic changes in the potential suitable distribution area

**DOI:** 10.3389/fpls.2022.1003368

**Published:** 2022-11-23

**Authors:** Yiheng Wang, Jiahui Sun, Ping Qiao, Jingyi Wang, Mengli Wang, Yongxi Du, Feng Xiong, Jun Luo, Qingjun Yuan, Wenpan Dong, Luqi Huang, Lanping Guo

**Affiliations:** ^1^ State Key Laboratory Breeding Base of Dao-di Herbs, National Resource Center for Chinese Materia Medica, China Academy of Chinese Medical Sciences, Beijing, China; ^2^ Key Laboratory of Biology and Cultivation of Herb Medicine, Ministry of Agriculture and Rural Affairs, Beijing, China; ^3^ Kunming Xishan Forestry and Grassland Comprehensive Service Center, Kunming, China; ^4^ Laboratory of Systematic Evolution and Biogeography of Woody Plants, School of Ecology and Nature Conservation, Beijing Forestry University, Beijing, China

**Keywords:** *Coptis*, plastid genetic diversity, divergence time, biogeographic history, potential suitable distribution, phylogenetic relationship

## Abstract

The genus *Coptis* belongs to the Ranunculaceae family, containing 15 recognized species highly diverse in morphology. It is a conspicuous taxon with special evolutionary position, distribution pattern and medicinal value, which makes it to be of great research and conservation significance. In order to better understand the evolutionary dynamics of *Coptis* and promote more practical conservation measures, we performed plastome sequencing and used the sequencing data in combination with worldwide occurrence data of *Coptis* to estimate genetic diversity and divergence times, rebuild biogeographic history and predict its potential suitable distribution area. The average nucleotide diversity of *Coptis* was 0.0067 and the hotspot regions with the highest hypermutation levels were located in the *ycf1* gene. *Coptis* is most likely to have originated in North America and Japanese archipelago and has a typical Eastern Asian and North American disjunct distribution pattern, while the species diversity center is located in Mid-West China and Japan. The crown age of the genus is estimated at around 8.49 Mya. The most suitable climatic conditions for *Coptis* were as follows: precipitation of driest quarter > 25.5 mm, annual precipitation > 844.9 mm and annual mean temperature -3.1 to 19 °C. The global and China suitable area shows an upward trend in the future when emission of greenhouse gases is well controlled, but the area, especially in China, decreases significantly without greenhouse gas policy interventions. The results of this study provide a comprehensive insight into the *Coptis* evolutionary dynamics and will facilitate future conservation efforts.

## Introduction

Understanding the evolutionary dynamics of living organisms is central to characterize biodiversity on Earth (Dong et al., 2022). The study of taxa with a significant phylogenetic position is a research hotspot in evolutionary biology. Ranunculaceae, located in the early-diverged of eudicot, has fascinated botanists for decades due to its fantastic features in diversification, evolution, and phylogeny (Zeng et al., 2014; Wei et al., 2019). In addition, intercontinental disjunction distribution is another research hotspot that has attracted considerable attention from botanists and biogeographers (Duan et al., 2020; Song et al., 2020; Nge et al., 2022). Understanding the past disjunct distribution pattern, timing, and the associated drivers is also a critical step in elucidating a clear evolutionary story. The Eastern Asian and North American (EA-NA) distribution is one of the most well-known disjunct distribution patterns, and the Bering land bridge probably provided opportunities for floristic exchange (Feng et al., 2021; Ye et al., 2022). Distribution of species was significantly shaped by environmental conditions, including temperature and precipitation. As a result, modeling species distribution has become popular in conservation, ecology, biogeography and evolution studies (Li et al., 2020; Huang et al., 2020).

*Coptis*, belongs to the Ranunculaceae family, which contains 15 recognized species that are highly diverse in floral morphology, but most species are extremely endangered, and disjunctly distributed from the warm-temperate to the boreal zone of East Asia and North America (Tamura, 1995). The species of this genus are also important medicinal plants worldwide. ‘Huanglian’, the rhizome of *Coptis* plants, has been clinically used as an antiviral, antimicrobial and anti-inflammatory agent for thousands of years (He et al., 2014). Currently, as the COVID-19 pandemic sweeps the world, ‘Huanglian’ shows the potential for fighting against the epidemic (Ma et al., 2020; Xu et al., 2022). In general, *Coptis* has a special evolutionary position, typical disjunct pattern and important medicinal value, which makes it to be of great research and conservation significance. To better characterize this special genus, an updated understanding of its evolutionary history, through a robust study on genetic variation, phylogeny, divergence time, and past disjunct distribution pattern is required. In addition, it is also necessary to develop more practical conservation measures, and predict its potential suitable distribution area from present to future.

As a well-known Chinese herbal medicine, the synthetic pathways of alkaloids derived from *Coptis* species, and their pharmacological and pharmacodynamic characteristics have been extensively studied (Minami et al., 2007; Yan et al., 2008; Chen et al., 2021; Liu et al., 2021; Yang et al., 2021). A few studies focused on phylogenetic reconstruction, distribution patterns assessment, divergence time estimation, and prediction of suitable distribution area (He et al., 2014; Xiang et al., 2016; Xiang et al., 2018; Li et al., 2020; Wang et al., 2020). Adequate sampling and sufficient variable molecular information are necessary for a reliable evolutionary process and prediction of the distribution. However, these studies only use a few DNA markers (*rbcL*, *matK*, *trnL-F*, *trnH-psbA*, ITS, etc.) which have limited variable information or the sample size of the studies was small that not all *Coptis* species from multiple distribution areas were included. Therefore, we expanded sampling to cover almost all the species of *Coptis* and used more informative markers to perform evolutionary research. In the meanwhile, we added more occurrence records to cover the worldwide distribution of *Coptis* for precise prediction of its suitable areas.

With the development of next-generation sequencing technologies, plastome genome sequences can be obtained more efficiently by directly sequencing total genomic DNA and *de novo* assembling whole plastid genomes (Sun et al., 2021). Due to its stable structure, rare recombination, moderate evolution rate, and largely uniparental inheritance, plastomes have been extensively used to reveal the evolutionary dynamics, such as phylogeny, phylogeography, demographic history and species diversity estimation (Aguirre Planter et al., 2020; Dong et al., 2021a; Dong et al., 2021b; Wang Y. et al., 2021; Wang Z. et al., 2021).

In this study, we newly sequenced, assembled eleven plastomes and analyzed twelve plastomes of *Coptis* (one of which was downloaded from GenBank). Combined with the geographical and climatic data of *Coptis* global distribution area, we aimed to (1) evaluate the plastome variation and identify the most variable regions, (2) reconstruct the phylogenetic relationship and estimate divergence times, (3) rebuild the biogeographic history and infer the formation of their disjunct pattern, and (4) predict its potential suitable distribution area. By combining these aspects, we aim to elucidate the evolutionary dynamics of this important genus and propose more reasonable protection measures.

## Material and methods

### Plant material and DNA extraction

Eleven specimens of genus *Coptis* were obtained from the herbarium of PE (Herbarium, Institute of Botany, CAS, Beijing, China) and CMMI (Institute of Chinese Materia Medica, China Academy of Chinese Medical Sciences, Beijing, China) (**Table S1**). The sequence of *C. japonica* was downloaded from GenBank (NC054329). Total genomic DNA was extracted from specimens using a modified cetyl trimethyl ammonium bromide (CTAB) method and purified with the Wizard DNA clean-up kit (Promega Corporation, Madison, WI, USA) (Li et al., 2013). DNA quality was assessed by spectrophotometry and stored at the -20°C.

### Plastome sequencing, assembly, and annotation

After fragmenting into 300-350 bp fragments by sonication, a pair-end library was constructed using the NEBNext Ultra™ DNA library prep kit (New England Biolabs, Ipswich, MA, USA). Pair-end (PE150) sequencing of 11 accessions was performed on the Illumina HiSeq XTen platform at Novogene Co., Ltd (Beijing, China). The raw data of the PE150 sequencing were filtered using the Trimmomatic 0.39 software (with settings: ILLUMINACLIP : TruSeq3-PE. fa:2:30:10:1:true LEADING:20 TRAILING:20 SLIDINGWINDOW:4:15) (Bolger et al., 2014). *De novo* assembly of the high-quality reads was performed using the Getorganelle v1.7.5 software with the suggested settings: -R 15 and -k 85,105 (Jin et al., 2020). Ultimately, all reads were mapped to the assembled plastome sequence to verify the assembly accuracy in Geneious 8.1 software (Biomatters Ltd., Auckland, New Zealand) as a double-check process. Gene annotation was performed using the online platform CPGAVAS2 with default settings and manual checking in Sequin to avoid missing or incorrectly annotated genes (Shi et al., 2019). The circle maps of the plastomes were plotted using the online program Chloroplot (https://irscope.shinyapps.io/Chloroplot/).

### Comparison of plastomes

Twelve plastomes were aligned using the multiple alignment software MAFFT online (https://mafft.cbrc.jp/alignment/server/) and manually adjusted using Se-al 2.0. Comparison of the whole plastomes of *Coptis* species was performed using the mVISTA program (http://genome.lbl.gov/vista/mvista/submit.shtml) in the Shuffle-LAGAN mode with *C. aspleniifolia* as a reference. Additionally, the nucleotide diversity (Pi, π) and Indels were calculated based on a 500-bp sliding window using the DnaSP v5.10 software (Librado and Rozas, 2009). Circos analysis was performed on the indel and nucleotide diversity data using the OmicStudio tools (https://www.omicstudio.cn/tool/) to visualize the hotspot region. A line graph was plotted to show the hyper-mutation region in detail (Gu et al., 2014).

### Phylogeny, biogeography and divergence time estimation

A total of 19 plastome sequences were used to reconstruct the phylogeny, including the *Coptis* species and seven outgroup plastomes from GenBank (**Table S1**). All genome sequences were aligned using the MAFFT software and ambiguous regions were trimmed by the Gblocks 0.91b program (Castresana, 2000). The program ModelFinder was used to select the best-fit model according to the Bayesian information criterion (Kalyaanamoorthy et al., 2017). The maximum likelihood tree was inferred using IQ-TREE with the TVM+F+R3 model and 5,000 ultrafast bootstraps in PhyloSuite (Nguyen et al., 2015; Zhang et al., 2020). Bayesian Inference phylogenies were inferred using MrBayes 3.2.6 (Ronquist et al., 2012) under GTR+I+G+F model (12 parallel runs, 500,000 generations), in which the initial 25% of sampled data were discarded as burn-in. Trees were visualized in FigTree v1.3.1.

Divergence time estimation was performed using a relaxed log normal clock model in the BEAST v2.6.6 platform, using the GTR substitution model and a speciation Yule Process tree prior (Bouckaert et al., 2019). The Markov chain Monte Carlo chains (MCMC) were run for 900 million generations and sampled every 1,000 generations with a sampling frequency of 1,000 generations. Secondary calibration points for dating are listed in **Table S2** according to previous studies (Xiang et al., 2018; Wei et al., 2019). The adequate effective sample size values (ESS > 200) were checked in Tracer 1.6. After a burn-in of 25%, a maximum clade credibility (MCC) tree with 95% highest posterior density intervals on each node was calculated using TreeAnnotator 2.1.3 and displayed in FigTree v1.3.1.

Based on the present distribution, we delimited four biogeographical areas: A, China mainland; B, Taiwan island; C, Japanese archipelago and a part of the Russian Far East; D, North America. We estimated ancestral distributions using the R package BioGeoBEARS implemented in Reconstruct Ancestral State in Phylogenies (RASP 4.0) (Yu Y. et al., 2020). The Dispersal Extinction Cladogenesis model with the jump dispersal parameter (DEC+J) was taken as the best model according to the model test in RASP (**Table S3**). In addition, we showed only the most likely status (MLS) for nodes where dispersal or vicariance had occurred.

### *Coptis* occurrences and species diversity

The worldwide occurrence records of *Coptis* species were collected from the Global Biodiversity Information Facility (GBIF; occurrence download https://doi.org/10.15468/dl.zqzb7b) and the National Plant Specimen Resource Center (NSII; http://www.nsii.org.cn/). Two-step approach for distribution records quality control was carried out. First, checking the species name in The Plant List (http://www.theplantlist.org/), removed unresolved or incorrect species records and corrected synonyms species. Second, generating the respective distribution maps for each species in ArcGIS 10.8, manual check the points inconsistent with the flora description and verified the species credibility. After removing or revising distribution points, 9,182 distribution records of *Coptis* species were obtained (**Table S4**). The global land area was divided into 2° grid cell by ArcGIS and the number of species per grid were counted to determine the species diversity of *Coptis*. A density map was plotted to visualize the distribution pattern of *Coptis* species diversity.

### Climatic variables and distribution modeling

In order to reduce sampling deviation in suitable habitats predicted by the MaxEnt model, we performed spatial rarefying of the data obtained in the previous step on a 10 km resolution using the SDM toolbox v2.5 and 2,898 records left for MaxEnt modeling (Brown et al., 2017).

A total of 19 climatic variables with a spatial resolution in 2.5’ for current climate data (average for 1970 - 2000) and four future periods data (BCC-CSM2-MR for 2021-2040, 2041-2060, 2061-2080, 2081-2100) used in the prediction of suitable species distributions were downloaded from WorldClim version 2.1 (https://www.worldclim.org/). We chose two scenarios, namely SSP126,and SSP585, to represent two extreme conditions in the future: a scenario with greenhouse gas well controlled at a low concentration (SSP126, 2.6 W/m2 in 2100) and a scenario with global warming trend without climate policy intervention (SSP585, 8.5 W/m2 in 2100), respectively (Zhao et al., 2021). All the climatic variables were converted to ASCII format using ArcGIS 10.8. To minimize overfitting of the MaxEnt model and ensure prediction accuracy, a Pearson correlation analysis was performed among the 19 climatic variables using the R package *ggpairs* (Emerson et al., 2013), and highly corelated ones were removed (> 0.75, **Figure S1**) (Dakhil et al., 2019). The correlation of climatic variables was tested all the species together, because one of our targets is to predict the whole genus potential suitable habitat and to raise the protection measures for *Coptis*. Ultimately, by using the software MaxEnt 3.4.1, eleven climatic variables were selected and entered along with the spatial rarefied occurrence data to predict potentially suitable distribution of *Coptis* species.

We set the cross-validation method to randomly selected 75% sites for model training, and the remaining for validation; the model was trained for ten replicate runs (Zhang Y. et al., 2021). Other parameters were kept as the default settings. The calculation result of the MaxEnt model was a grid layer in ASCII format and the value in each grid represented the potential suitable rate of species varying from 0 to 1. Then, we loaded the result into ArcGIS 10.8 to visualize the map of the potential species distribution and regrouped them into four levels as previous studies did: no suitability (0-0.2); low suitability (0.2-0.4); medium suitability (0.4-0.6); and high suitability (0.6-1) (Abolmaali et al., 2018; Zhang et al., 2018; Xu et al., 2019; Kong et al., 2021). Model calibrations and robustness validation were evaluated using the area under the curve (AUC) of the receiver operating characteristics (ROC) curve. The AUC value ranged from 0 to 1 with the following evaluation criteria: poor (0.6–0.7), fair (0.7–0.8), good (0.8–0.9), and excellent (0.9–1) (Zhao et al., 2021). In addition, the contribution rate and jackknife test generated by the MaxEnt model were used to measure the contribution weights of eleven climatic variables. The tool “Distribution changes between binary SDMs” in SDM toolbox v2.5 was used to calculate the changes in distribution area between adjacent time periods.

## Result

### Features and variation of plastomes genomes

Eleven *Coptis* plastomes were obtained by *de novo* assembly, and deposited in GenBank or National Genomics Data Center (NGDC) with the accession numbers listed in **Table S1**. The genomes ranged from 153,959 to 154,932 bp in size and contained 113 unique genes (80 protein coding genes, 29 tRNA genes, and four rRNA genes). The whole plastomes had a typical quadripartite structure including a pair of inverted-repeats (IR) regions (26,074 - 26,225 bp), large single copy (LSC) regions (84,112 - 85,178 bp) and small single copy (SSC) regions (17,215 - 17,606 bp). The average GC content ranged from 38.2 to 38.3%.

**Figure 1:**
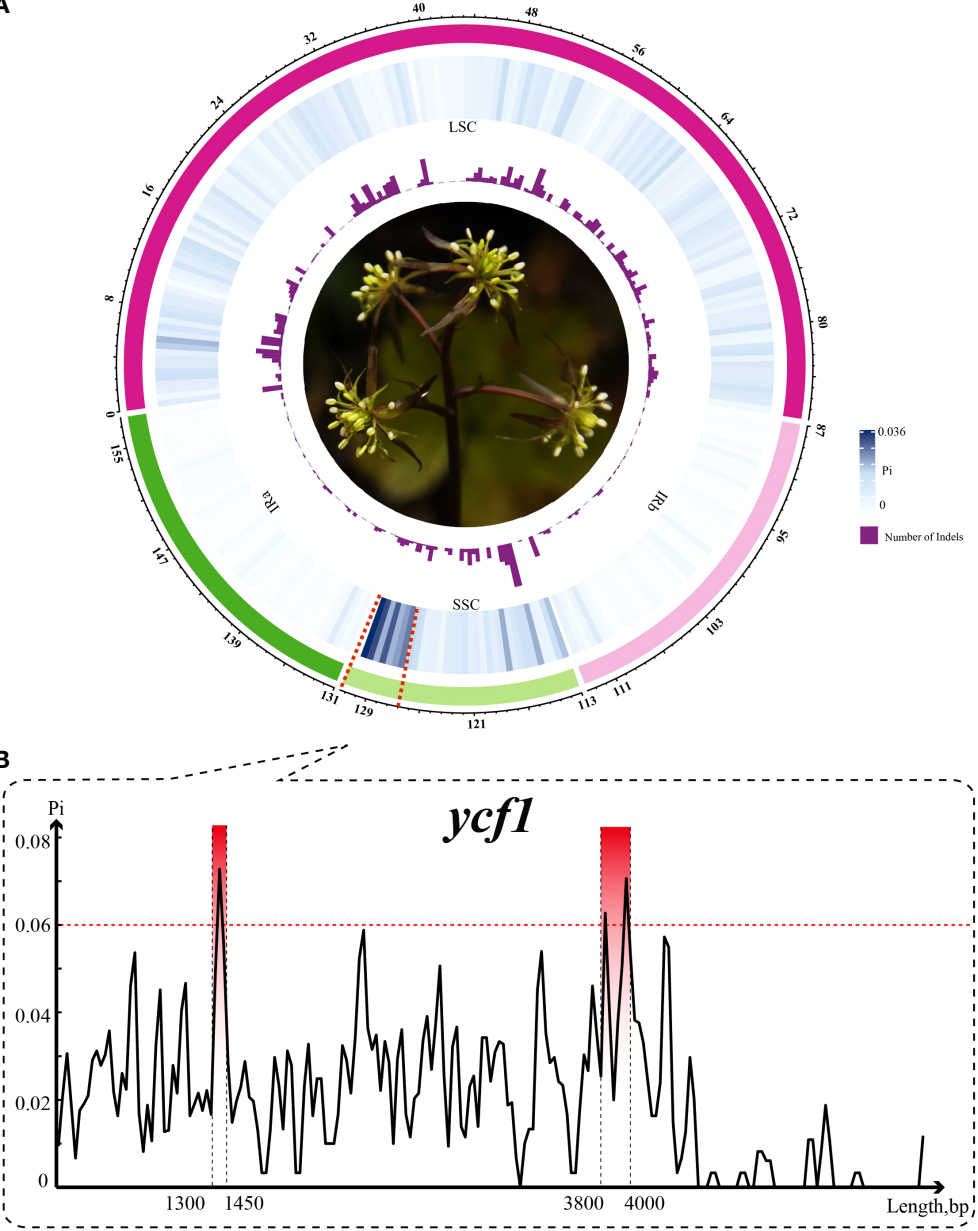
Variation of *Coptis* plastomes. **(A)** Circos plot showing the indel and nucleotide diversity of *Coptis*. Circles from the outer to inner area show the following: structure of plastomes indicated by different colors, nucleotide diversity shown by a heatmap and indel count shown by a histogram. Window size of each grid is 500 bp. **(B)** Sliding-window analysis of the *ycf1* gene. The most divergent region (top three region with Pi > 0.06) is indicated.

The results from the mVISTA program analysis showed that most mutation events occurring in the spacer regions and gene of the plastomes were highly conserved, especially in the exon regions (**Figure S2**). Total sequences had aligned length in 157,727 bp, which had 3,807 variable sites, 1,030 indels and the average nucleotide diversity was 0.0067. Based on the sliding window results, Pi and indels of *Coptis* species were visualized in a Circos map with a window size of each grid of 500 bp (**Figure 1A**). Most indels in the region were found in ndhF-rpl32. The Pi of a single window varied from 0 to 0.0359 and the indels of a single window varied from 0 to 40. Almost all mutations (92.5%) were located in the SSC and LSC regions, indicating highly conserved IR regions. Moreover, the most hypermutated region was located in the *ycf1* gene. In order to reveal the detailed information of this region, a new sliding-window analysis was performed with settings of 50 bp window length and 25 step size. The most divergent region (Pi > 0.06) of the *ycf1* gene was identified and limited to two tiny regions within 200 bp in size, considered as hotspots (**Figure 1B**). Compared to the three conventional plastid DNA barcodes *(trnH-psbA*, *rbcL* and *matK*), the two hotspots were nearly twice as high as the second highest barcode *trnH-psbA* and then followed by the complete *ycf1* (**Table 1**).

**Table 1:** The variability of the hypervariable markers and universal DNA barcodes.

Markers	Length	Variable sites		information sites		Nucleotide Diversity (Pi)
		Numbers	%	Numbers	%	
*rbcL*	1428	30	2.10	12	0.84	0.006
*matK*	1533	95	6.19	39	2.54	0.018
*trnH-psbA*	316	24	7.59	10	3.16	0.025
*rbcL + matK + trnH-psbA*	3277	149	4.55	61	1.86	0.012
*ycf1*	5772	405	7.01	197	3.41	0.020
*Two hotspots*	417	51	12.23	30	7.19	0.042

### Species distribution and diversity pattern

**Figure 2:**
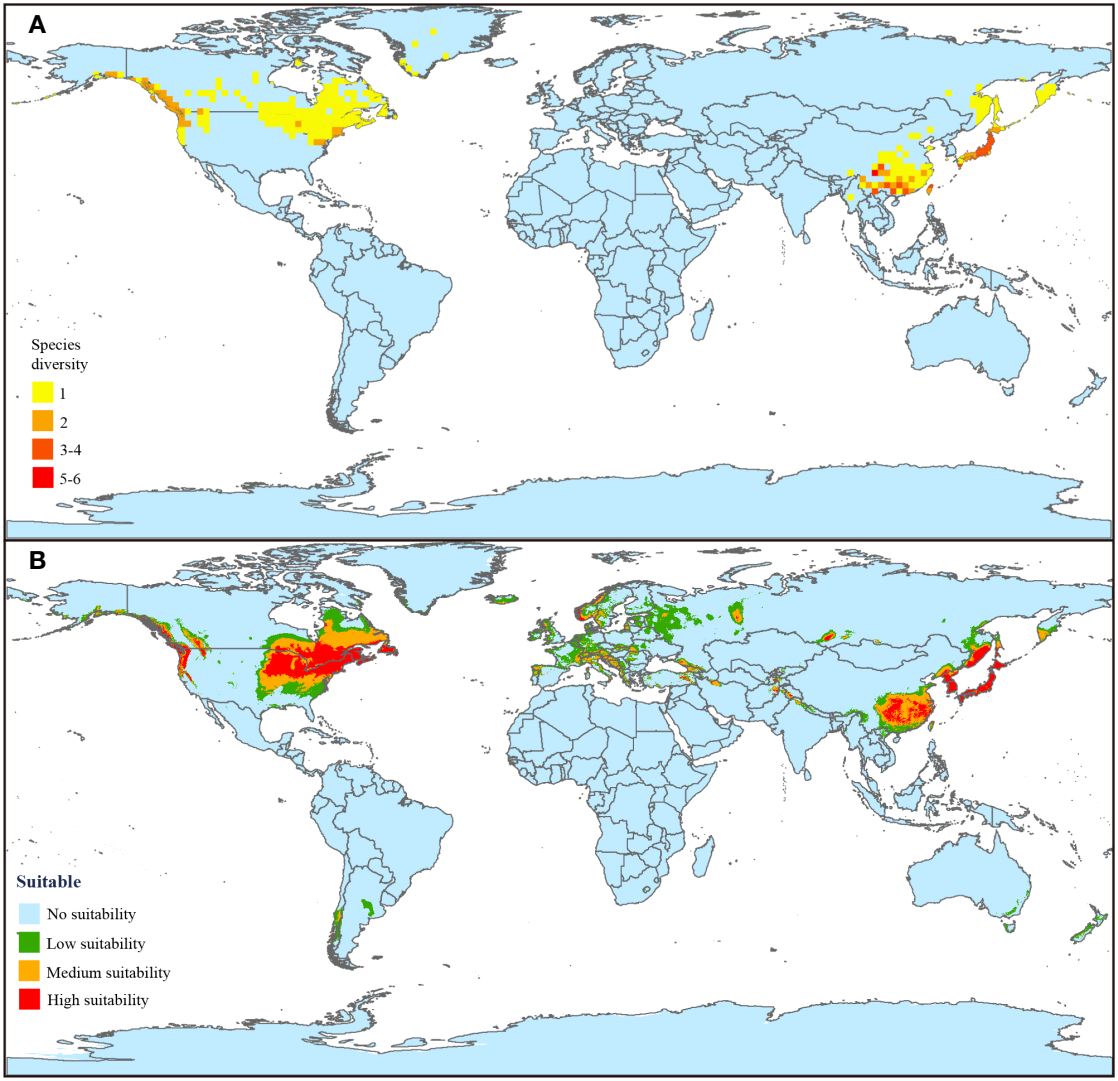
Current distribution and suitable habitat prediction of *Coptis*. **(A)** Current species distribution and diversity pattern. The global land area was divided into 2° grid cell by ArcGIS and the number of species per grid were counted to determine the species diversity of *Coptis*. The redder the grid, the higher the species diversity. **(B)** Current potential distribution of *Coptis* under 1970–2000 climate conditions. Four levels of suitability are shown in different colors as follows: no suitability (0-0.2, blue); low suitability (0.2-0.4, green); medium suitability (0.4-0.6, yellow); and high suitability (0.6-1, red).

All the 9,182 occurrence records consisted of 15 *Coptis* species that were mainly found in China, Japan, Canada, and United States of America, as well as additionally scattered in the Russian Far East, Korean Peninsula and Greenland (**Figure 2A**). This clearly indicated that *Coptis* has a typical Eastern Asian and North American (EA-NA) disjunct distribution pattern. The number of species per grid, a reflection of species diversity, varied from one to six. The grids contained more than three species all located in Eastern Asia, especially in Mid-West China and Japan, which could indicate that these two regions are diversity hotspots of *Coptis*. For North America, the regions along the Pacific Coast Range were slightly more diverse than other regions.

### Phylogeny, divergence time estimation and biogeographical history

**Figure 3:**
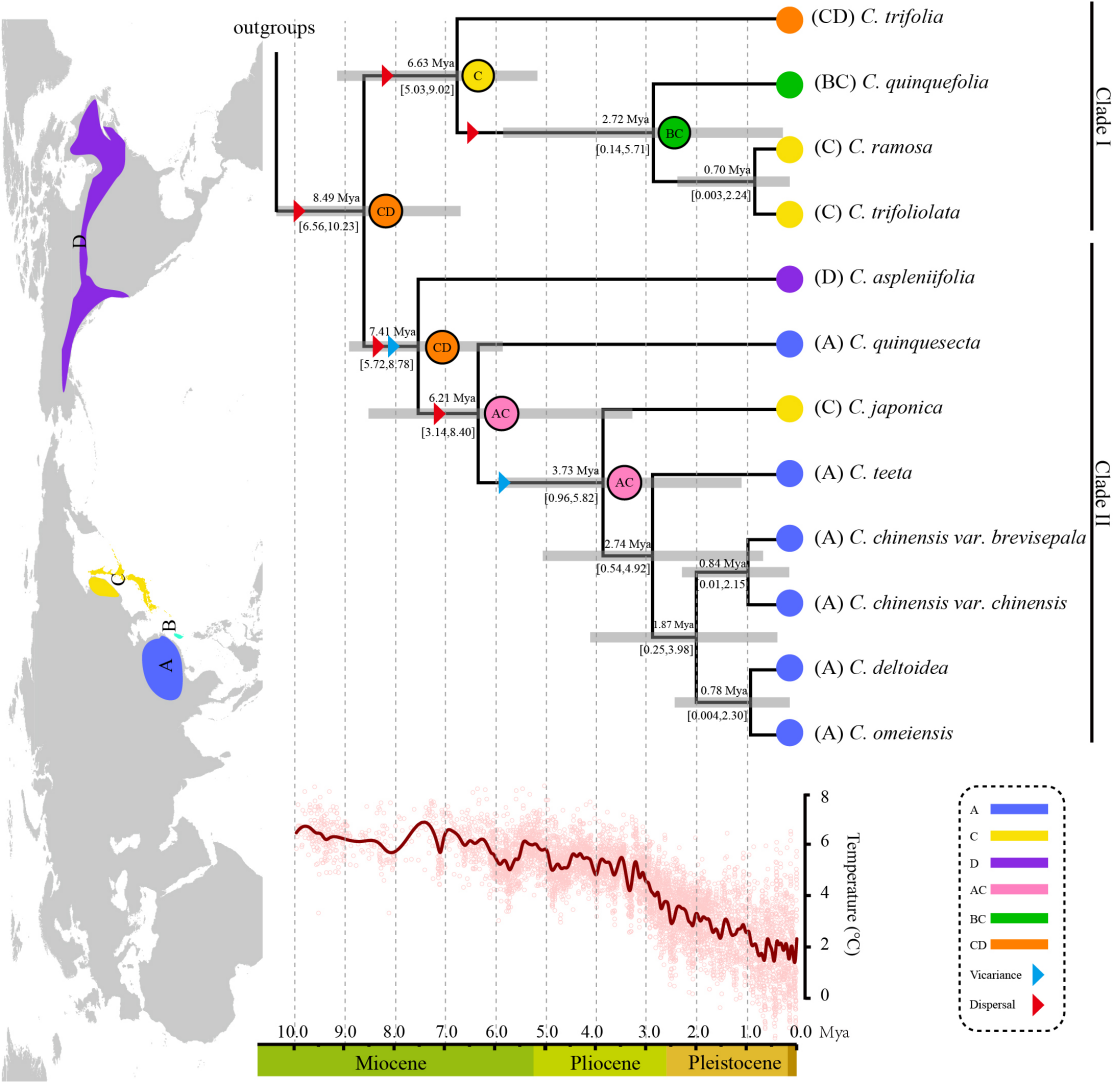
Combined dating analyses and ancestral habitat reconstruction of *Coptis*. The global temperature change in the past 10 Ma was obtained from Zachos et al. (Zachos et al., 2008). The insert Northern hemisphere map indicates the species distribution of *Coptis* used in the reconstruction with four defined biogeographical areas as follows: **(A)** mainland China; **(B)**, Taiwan island; **(C)**, Japanese archipelago and a part of the Russian Far East; **(D)**, North America. Dated phylogeny of *Coptis* was derived from **Figure S6**. Numbers above and under the branches indicate the mean divergence times and 95% confidence interval of each node, respectively. Blue bars indicate the 95% highest posterior density intervals. Letters and colors in the legend represent extant ancestral areas and combination of them. Pie chart labeled with letters at each node indicates the most likely ancestral area.

The 19 aligned sequences for phylogenetic reconstruction were 191,086 bp in length and 152,783 bp left after trimming by Gblocks. All the *Coptis* species formed a monophyletic group with 100% support. Apparently, *Coptis* species were divided into two large clades, namely Clade I and Clade II (**Figure 3** and **Figure S3**). Clade I (100% bootstrap value) comprised *C. aspleniifolia*, *C. quinquesecta*, *C. japonica*, *C. teeta*, *C. omeiensis*, *C. deltoidei*, *C. chinensis* var. *chinensis* and *C. chinensis* var. *brevisepala*. Species of Clade I were distributed disjunctively among three biogeographical areas (A, C and D). Clade II (100% bootstrap value) comprised *C*. *trifolia*, *C*. *quinquefolia*, *C. ramosa* and *C. trifoliolata*, and the latter two formed a monophyly. Additionally, Clade II also distributed disjunctively among three biogeographical areas (B, C and D).

According to the combined dating and RASP results, the *Coptis* genus was most likely to have originated in North America and the Japanese archipelago and the crown age was estimated to be at around 8.49 Mya (95% HPD: 6.56 – 10.23 Mya) in the late Miocene, when the two clades first diverged. In Clade I, *C. trifolia* first split off at around 6.63 Mya when a Bering Land Bridge dispersal (C & D) occurred. Then, the second dispersal (B & C) occurred at around 2.72 Mya when *C. quinquefolia* split off from *C. ramose* and *C. trifoliolata*. Species diversification in Clade II was gradual and successive. *C. aspleniifolia* diverged from Asian species to North American probably around 7.41 Mya. Moreover, *C. quinquesecta* and *C. japonica* successively separated at around 6.21 Mya and 3.73 Mya, corresponding respectively to a dispersal and a vicariance event that occurred between the Chinese mainland and Japanese archipelago with a part of the Russian Far East. This vicariance led to the colonization of *Coptis* in mainland China and the subsequent speciation into five species. In addition, these five species formed a subclade, in which *C. teeta* separated in 2.74 Mya, followed by a divergence of *C. chinensis* from its closest relatives (*C. deltoidei* and *C. omeiensis*) that occurred in 1.87 Mya.

### Modeling validation and dominant climatic variables

The MaxEnt model was used to simulate the suitable habitats of *Coptis*. The final ROC curve (shown in **Figure S4**), revealed that the average test AUC for the replicate runs was 0.883, with a standard deviation of 0.005, which indicated that the MaxEnt model performed efficiently and reliably in the prediction of suitable habitats.

The eleven selected climatic variables involved in potential suitable habitat prediction are listed in **Table 2**. The contribution weight of selected climatic variables is shown by the contribution rate and jackknife test result. The variable with the highest contribution rate was precipitation of the driest quarter (Bio 17, contribution rate 38.1%), followed by annual precipitation (Bio 12, contribution rate 37.4%) and annual mean temperature (Bio 1, contribution rate 10.2%). The jackknife test results revealed that the highest gain when used in isolation was still precipitation of the driest quarter (Bio 17, training gain 0.83), followed by annual mean temperature (Bio 1, training gain 0.80) and annual precipitation (Bio 12, training gain 0.79). It is undisputable that these three factors were the most dominant climatic variables in influencing the potential suitable habitat of *Coptis*, while the Bio 1 and Bio12 rankings are different by two assessment methods.

**Table 2:** The description and contribution weight of eleven selected climatic variables.

Variable	Description	Contribution rate	Jackknife test result
Bio1	Annual Mean Temperature	10.2	0.8
Bio2	Mean Diurnal Range (Mean of monthly (Max Temp - Min Temp))	0.2	0.17
Bio3	Isothermality (BIO2/BIO7) (×100)	7.4	0.47
Bio5	Max Temperature of Warmest Month	3.1	0.63
Bio7	Temperature Annual Range (Max Temp of Warmest Month - Min Temp of Coldest Month)	1.9	0.13
Bio8	Mean Temperature of Wettest Quarter	0.2	0.45
Bio9	Mean Temperature of Driest Quarter	0.4	0.63
Bio12	Annual Precipitation	37.4	0.79
Bio15	Precipitation Seasonality (Coefficient of Variation)	0.9	0.48
Bio17	Precipitation of Driest Quarter	38.1	0.83
Bio18	Precipitation of Warmest Quarter	0.2	0.67

The response curve of the three most dominant climatic variables shown in **Figure S5** was generated by the MaxEnt model and illustrates the quantitative relationship between the species presence probability and climatic variables and clearly elucidated the suitability conditions of *Coptis* under different climatic variables. According to the levels of high suitability (> 0.6), the corresponding climatic conditions should be suitable for species growth. In terms of the three dominant climatic variables, the optimum ranges of precipitation of the driest quarter should be more than 25.5 mm, annual precipitation should be more than 844.9 mm and annual mean temperature should range from -3.1 to 19.9°C.

### Current and future potential habitat prediction and dynamic changes

Based on the current climate condition (1970 - 2000) and occurrence records of *Coptis*, the global potential suitable habitat (suitable rate > 0.2) projected by the MaxEnt model was 1,315.0×10^4^ km^2^, which was mostly located in the northern temperate zone, and rarely occurred in tiny regions of New Zealand, Australia, Argentina and Chile (**Figure 2B**). In China, the potential habitat was 239.6×10^4^ km^2^, comprising 18% of global suitable area, whose area of the three suitable levels (suitable rate > 0.2) from high to low were 57.7×10^4^, 112.2×10^4^ and 69.7×10^4^ km^2^, respectively. The global potential high, medium, and low suitable areas covered 328.9×10^4^, 409.9×10^4^ and 576.2×10^4^ km^2^, respectively, and the high suitable area were restricted to the eastern border of the United States of America and Canada, the Pacific Coast Range, most of Northeast Asia, and Mid-West China.

**Figure 4:**
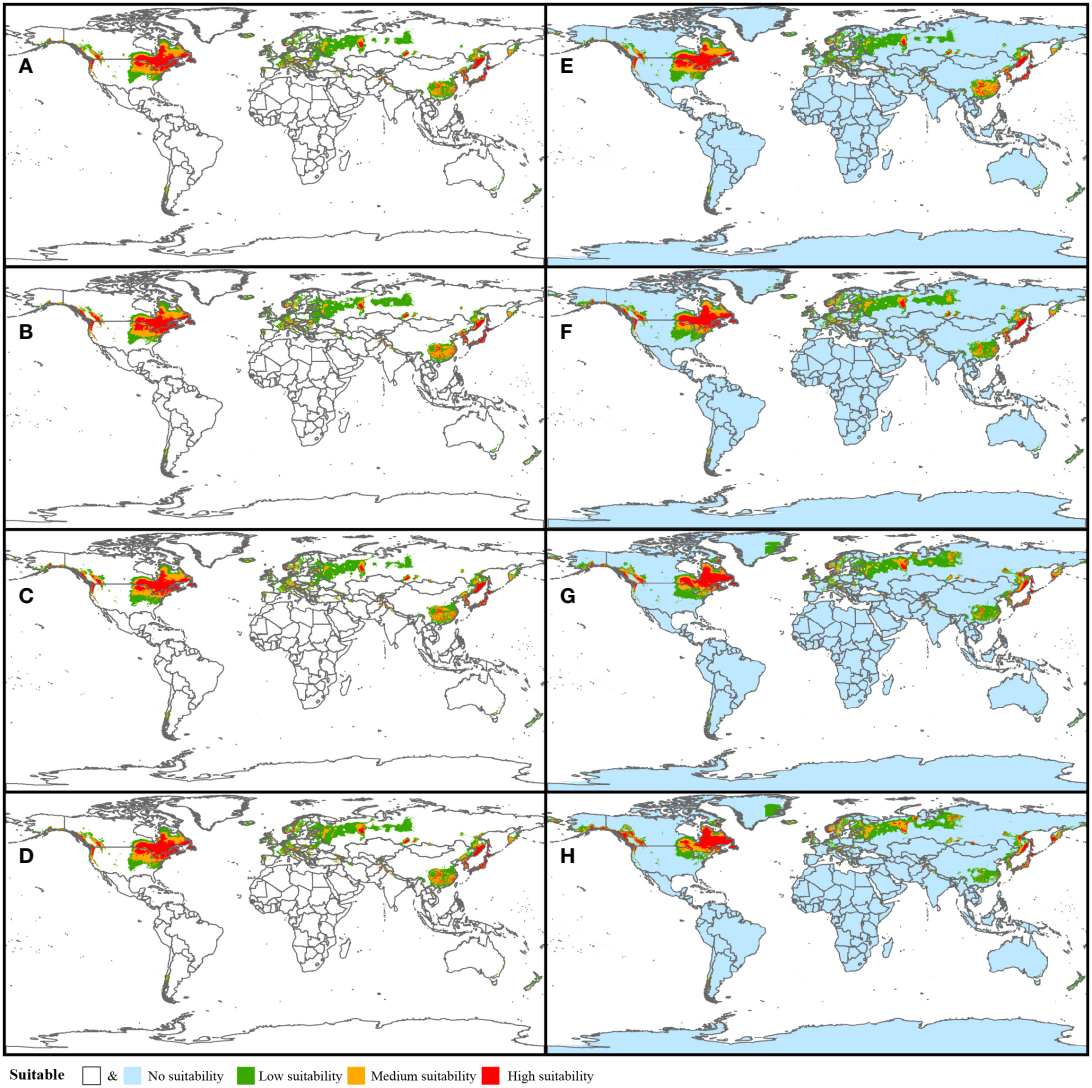
Potential habitat prediction of *Coptis* in the future periods: 2021-2040 (SSP126, **A** and SSP 585, **E**), 2041-2060 (SSP126, **B** and SSP 585, **F**), 2061-2080 (SSP126, **C** and SSP 585, **G**) and (SSP126, **D** and SSP 585, **H**).

**Figure 5:**
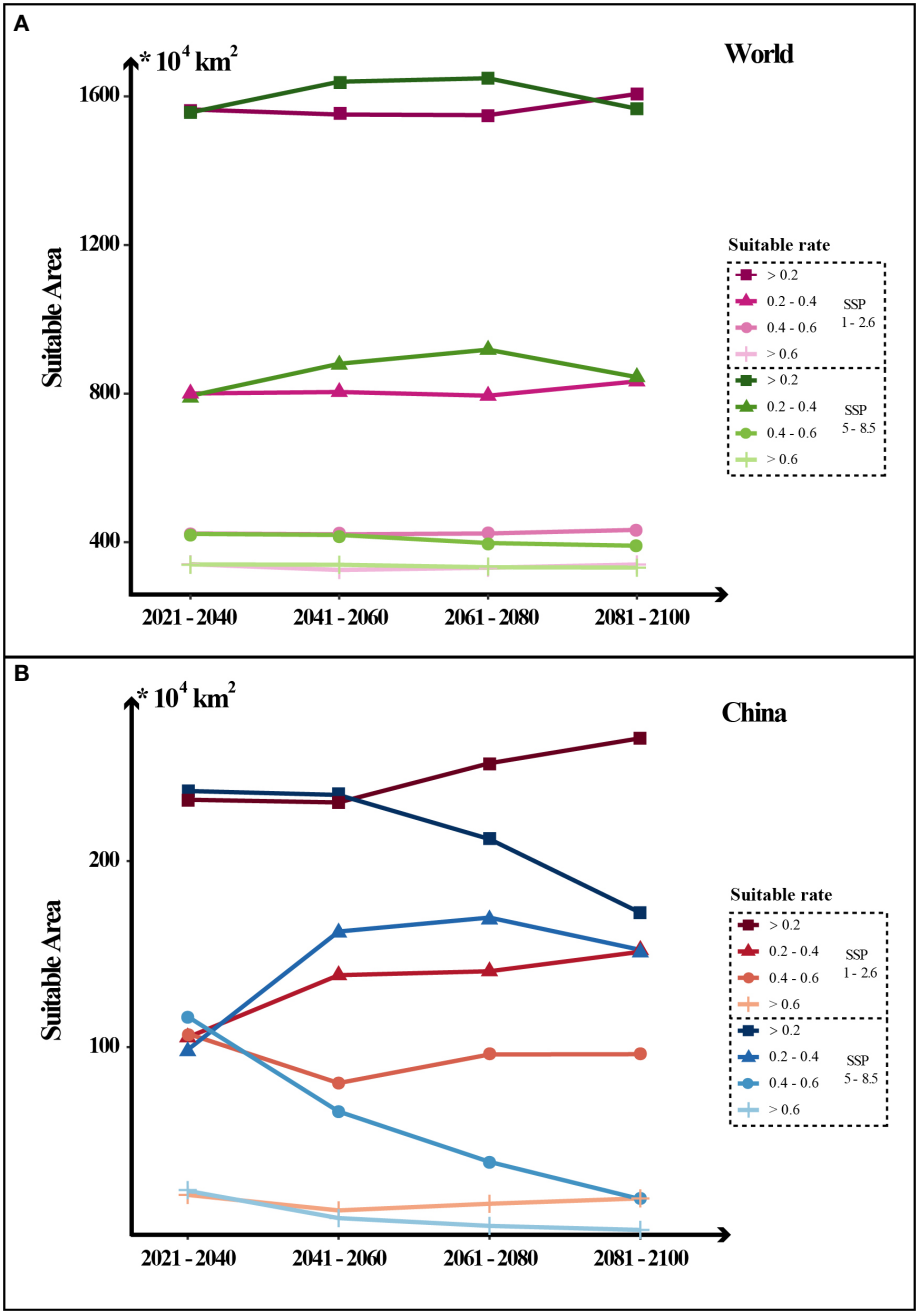
Dynamic changes in the area of potential *Coptis* suitable distribution in the world and China in the future four periods under two greenhouse gas emission scenarios (SSP126 and SSP585). The levels of suitability are shown in different line types: low suitability (0.2-0.4); medium suitability (0.4-0.6); high suitability (0.6-1) and total suitability (> 0.2, the sum of high, medium and low levels).

Two climate change scenarios (SSP585 and SSP126) were analyzed to predict the suitable habitat in the future four periods from 2021 to 2100 (**Figure 4**). As mentioned above, SSP585 is a scenario with no policy intervention and the radiative forcing will rise to 8.5 W/m^2^ by 2100. In this scenario, the global potential suitable area (suitable rate > 0.2) has an upward trend followed by a downward trend with the peak occurring at 1,648.8×10^4^ km^2^ (2061-2080). Meanwhile, the potential center noticeably shifts north. Correspondingly, the area in China declines continuously from 237.7×10^4^ km^2^ (2021-2040) to 172.3×10^4^ km^2^ (2081-2100). In particular, the high suitable area (suitable rate > 0.6) in China decreases most significantly, from 22.7×10^4^ km^2^ (2021-2040) to merely 1.7×10^4^ km^2^ (2081-2100). In the SSP126 scenario, the global warming trend may have been suppressed by 2100, the global suitable area shows a slight upward trend (**Figure 5**). The potential suitable area (suitable rate > 0.2) increases from 1,564.8×10^4^ km^2^ (2021-2040) to 1,606.4×10^4^ km^2^ (2081-2100) and the center of the suitable area is basically stable. In addition, in contrast to the SSP558 scenario, the suitable area in China shows a gradual upward trend, from 233.0×10^4^ km^2^ (2021-2040) to 266.1×10^4^ km^2^ (2081-2100). Also, the decline of the high suitable area (suitable rate > 0.6) is halted and rebounds to 18.6×10^4^ km^2^ (2081-2100). The shifts of distribution areas between adjacent time periods under two scenarios were vividly presented in **Figure S7**.

## Discussion

### Genetic divergence of *Coptis* species and candidate DNA barcodes

As mentioned above, *Coptis* is a genus with enormous value in research and development. Genetic diversity defines the evolutionary potential and resilience of species (Sun et al., 2021). Thus, it is crucially important to evaluate the genetic diversity of this genus. However, there is no comprehensive plastid genetic resource available. In this study, twelve *Coptis* plastomes were found to be highly conserved in genome structure and gene order, and no rearrangement occurred. Some variations were observed in the GC content and genome size. The GC content varied from 38.2 to 38.3% and the genome size varied from 153,959 to 154,932 bp, indicating the existence of genetic diversity. Compared to other medicinal plants, such as *Artemisia* (Pi = 0.0024), *Atractylodes* (Pi = 0.001), *Crataegus* (Pi = 0.00175) and *Ligusticum* (Pi = 0.002), *Coptis* (Pi = 0.0067) is a medicinal genus with relatively high genetic diversity (Kim et al., 2020; Wang Y. et al., 2021; Wu et al., 2022; Wei et al., 2022).

The mVISTA results revealed that, under evolutionary constrains and natural selection pressure, the noncoding region was more variable than the coding region (Zhang H. et al., 2021). According to the sliding-window analysis and Circos map, the LSC and SSC regions were more variable than the IR regions and may be possibly due to copy corrections between IR sequences by gene conversion (Zhao et al., 2018). In addition, it also revealed that the mutations were not uniformly or randomly distributed across all regions of the plastome. The aggregation of mutations in certain regions creates hotspots. Hotspots are potential DNA barcode development region that are usually efficient for interspecies discrimination and phylogeny reconstruction (Downie and Jansen, 2015; Song et al., 2017; Pang et al., 2019). Clearly, the hotspot regions of *Coptis* were located in the junction of SSC and IRs, which belonged to the *ycf1* gene. As the second largest gene in the plastid genome, *ycf1* is recognized for its variability in seed plants (Dong et al., 2015). Since it is very long (more than 5 kb) and quite variable in application, two highly mutant regions, namely *ycf1*a (800 bp) and *ycf1*b (1100 bp), of *ycf1* have been developed and used as the most promising plastid DNA barcodes (Dong et al., 2012). Coincidentally, in this study, the precise locations of two hotspots in the *ycf1* were identified, which corresponded to the *ycf1*a and *ycf1*b mutant regions, respectively. Furthermore, the size of these two hotspots were limited to 200 bp. Compared to the three conventional plastid DNA barcodes (*trnH-psbA*, *rbcL* and *matK*), these two regions are much shorter in size but more informative, which qualifies them as mini-barcodes. Notably, mini barcodes may facilitate discrimination of DNA degradation materials, such as herbarium specimens, processed medicinal plant, or even fossil material. Moreover, this strategy of developing taxon-specific barcodes by comparing plastid genome sequences has also been applied in other medicinal plant taxa, including *Panax*, *Senna*, and *Paeonia* (Dong et al., 2014; Yu X. et al., 2020; Yang et al., 2022). Collectively, these two hotspots should be promising mini-barcodes for *Coptis* species identification in future applications.

### Phylogenetic inferences and evolutionary history

Elucidation of the phylogenetic relationship of *Coptis* is crucial in understanding its evolutionary history. In previous studies, the phylogeny reconstruction of *Coptis* was performed using a small number of DNA loci, such as *trnL-F*, *trnD-T*, *trnH-psbA*, *rpoB*, *accD*, *rbcL*, or limited sampling, which resulted in inconsistent phylogenetic relationships, especially for the four late-diverging species: *C. deltoidea*, *C. omeiensis*, *C. chinensis* var. *chinensis* and *C. chinensis* var. *brevisepala* (He et al., 2014; Xiang et al., 2016; Xiang et al., 2018; Wang et al., 2020). Xiang et al. (2016) suggested that *C. chinensis* var. *brevisepala* formed a clade with *C. deltoidea* and *C. omeiensis*, while *C*. *chinensis* var. *chinensis* was separated in the early-diverged position based on the matrix combining *trnL-F*, *trnH-psbA* and ITS. Also, Wang et al. indicated that *C. chinensis* var. *brevisepala* was in the early-diverged part of these four species based on the dataset comprising *trnH-psbA*, *rbcL* and *matK*. Importantly, based on adequate sampling and whole plastid genomes with adequate genetic information, this study revealed the most comprehensive phylogeny for *Coptis*. The robust relationship was reconstructed and these controversial branches were fully resolved.

The evolutionary history was clearly shown by estimating the divergence time and biogeography of *Coptis*. The species-level age estimation suggested a crown group age of 8.49 Mya (95% HPD: 6.56 – 10.23 Mya) in the late Miocene, which was largely consistent with previous results with an age of 9.55 Mya (95% HPD: 6.66–12.92 Mya) determined by DNA barcodes (Xiang et al., 2018). In the middle to late Miocene, the most significant events influencing the evolutionary patterns of global plants were the uplift of the Qinghai-Tibetan Plateau in East Asia and the Rocky Mountains in western North America. These two significant tectonic events in the eastern and western hemispheres had effects on global atmospheric circulation, weathering rates, monsoon and even riverway trend, which might be the main factors underlying the origin and diversification of *Coptis* (Qiu et al., 2011; Pound et al., 2011). *Coptis* probably originated in the Japanese archipelago and a part of the Russian Far East (area C) and North America (area D), and the current distribution of *Coptis* seems to have been shaped by several dispersal and vicariance events. The Bering Land Bridge was the most likely dispersal corridor between the two continents, which has been considered as an explanation for the disjunct distribution of related extant floras in EA and NA. Furthermore, sea level fluctuations and climate cooling since the Pliocene have provided abundant opportunities for promoting dispersal and vicariance among the three Asian biogeographical areas and speciation of *Coptis* occurred rapidly since that time (Haq et al., 1987; Qian and Ricklefs, 2004; Qiu et al., 2011; Xiang, 2020).

It is well accepted that the climatic oscillations, sea-level fluctuations and land bridge configuration promoted speciation and extinction, shaped distribution and diversity pattern of species (Qian and Ricklefs, 2000; Qiu et al., 2009). The diversity center of *Coptis* is located in Japan and the mid-west of mainland China, which are the major parts of the widely recognized biodiversity hotspots “Sino-Japanese Floristic Region” (Hanson et al., 2010). Moreover, for disjunct distributed taxa, diversity is generally higher in East Asia than in North America. The main reason is that East Asia was less influenced by ice sheets during the Quaternary period thus having a lower species extinction rate, and older and more complex topographical features than North America, which may create this biodiversity hotspot in the world with much more species diversity than that in North America (Qian, 2002).

### Conservation implications for *Coptis*

Temperature and precipitation are regarded as two of the main variables restricting the range of the majority of terrestrial plant species, as well as that of *Coptis*. As shown by the contribution weight of dominant climatic variables in the results, precipitation is one of the most important factors in future conservation. Understanding the suitable climatic ranges and trend of changes in potential suitable habitat for *Coptis* (precipitation of driest quarter > 25.5 mm, annual precipitation > 844.9 mm and annual mean temperature -3.1 to 19°C), may contribute to provide a basis for ex-situ conservation strategies and the establishment of ex-situ resource nursery for *Coptis* in the future and will also be useful for guiding cultivation and introduction of domestication.

Currently, global climate change greatly affects the distribution of various species and it is also a challenge for all of humanity. Under two greenhouse gas emission scenarios (SSP126 and SSP585), the suitable distribution area of *Coptis* varies largely. In the low-emission condition, the suitable area (suitable rate > 0.2) in world is stable or even shows a slight upward trend, and it is increasing significantly in China. However, without climate policy intervention, the global suitable (suitable rate > 0.2) distribution moves northward and the area in China precipitously declines. The results demonstrate the necessity of tight regulation of greenhouse gas emissions. Confronted by the challenge of climate change, it is important for the state parties and signatories to fulfill the Paris Agreement to reduce their carbon output. These actions should be beneficial not only for the *Coptis* conservation, particularly in China, but also for all lives in the world.

## Data availability statement

The datasets presented in this study can be found in online repositories. The names of the repository/repositories and accession number(s) can be found in the article/**Supplementary Material**.

## Author contributions

YW and JS wrote and revised the manuscript. PQ participated in the experiments. JW, YD, JL, QY, YW, MW and FX collected the materials and analyzed the data. WD, LH and LG conceived and designed the research. All authors contributed to the article and approved the submitted version.

## Funding

This research was funded by CACMS innovation Fund (No.CI2021A03909), National Key Research and Development Program of China (2017YFC1703700: 2017YFC1703704), National Natural Science Foundation of China (No.81891014 & No.81874337), Innovation Team and Talents Cultivation Program of National Administration of Traditional Chinese Medicine (No. ZYYCXTD-D-202005) and Genetic Resources Management Project of State Forestry and Grassland Administration (KJZXSA202105).

## Acknowledgments

The authors would like to thank professor Shiliang Zhou, Dr. Chao Xu, Kangjia Liu and Enze Li for providing suggestions, and thank the DNA Bank of China in Institute of Botany, Chinese Academy of Sciences for providing materials.

## Conflict of interest

The authors declare that the research was conducted in the absence of any commercial or financial relationships that could be construed as a potential conflict of interest.

## Publisher’s note

All claims expressed in this article are solely those of the authors and do not necessarily represent those of their affiliated organizations, or those of the publisher, the editors and the reviewers. Any product that may be evaluated in this article, or claim that may be made by its manufacturer, is not guaranteed or endorsed by the publisher.
