# Supplementary figures and images for: Evolutionary history of genus Coptis and its dynamic changes in the potential suitable distribution area
Source: Front Plant Sci. 2022 Nov 23;13:1003368. doi: 10.3389/fpls.2022.1003368 (PMC9727247; doi:10.3389/fpls.2022.1003368)

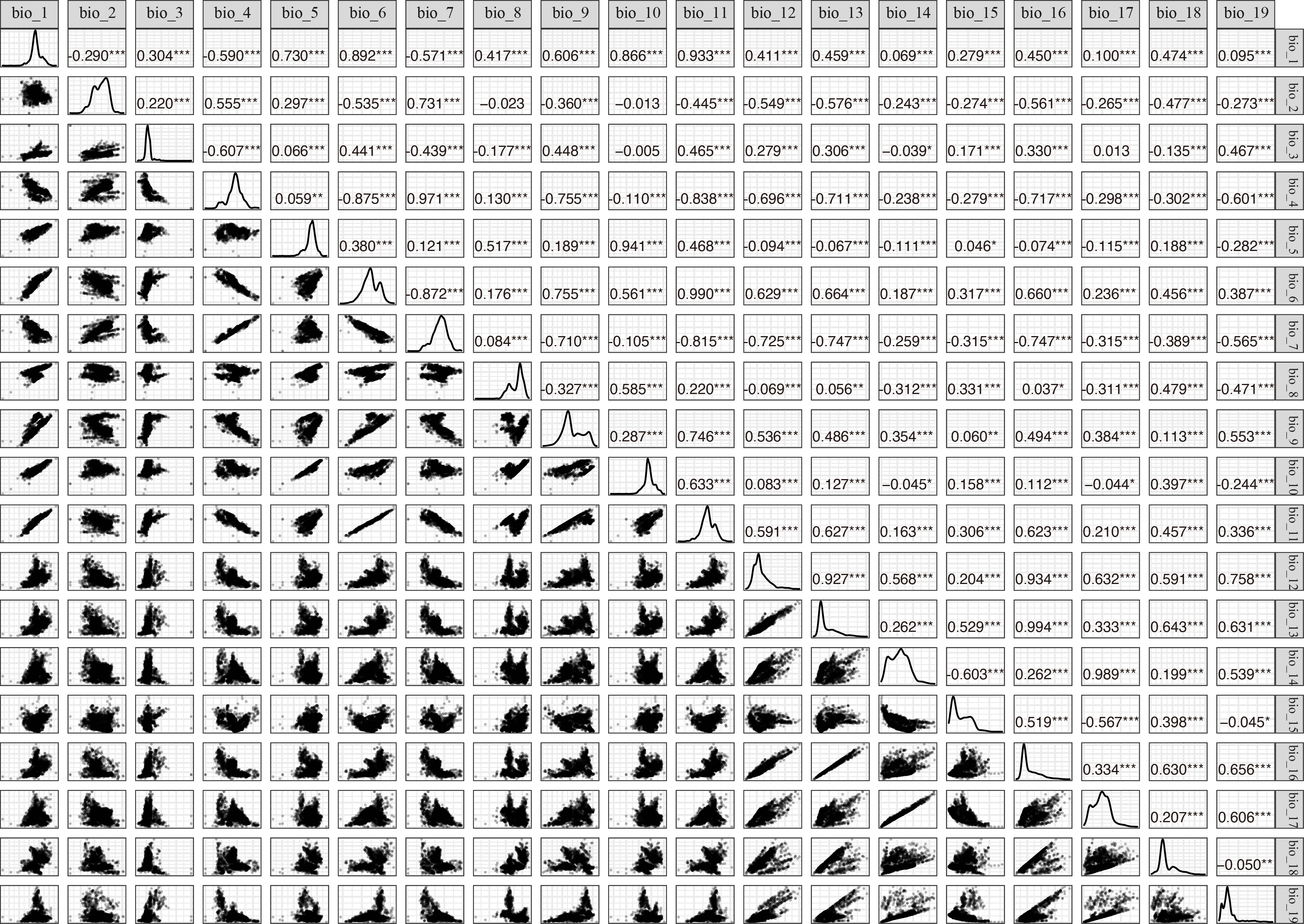

Supplement: Supplementary file 1 [file Image_1.jpeg]

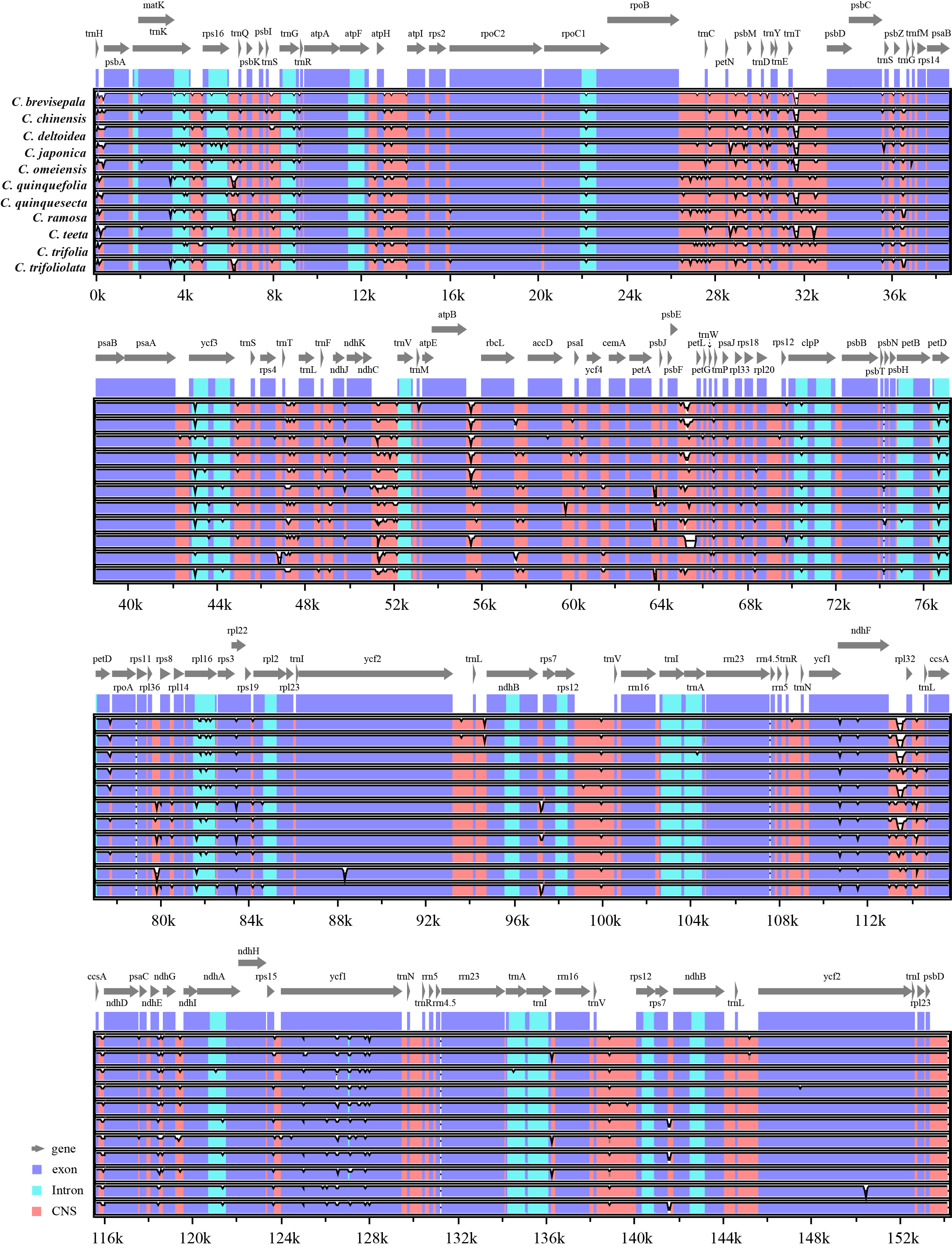

Supplement: Supplementary file 2 [file Image_2.jpeg]

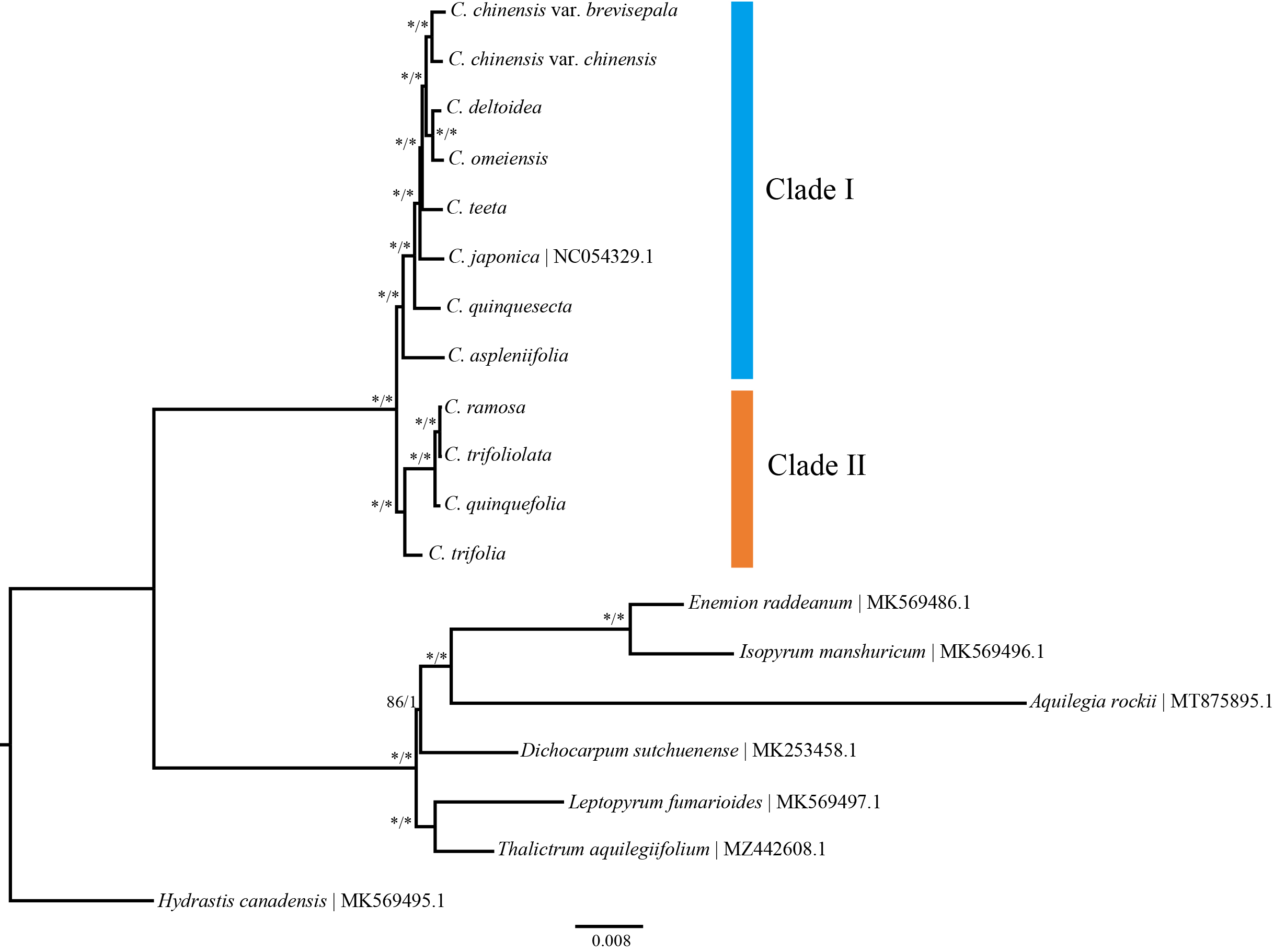

Supplement: Supplementary file 3 [file Image_3.jpeg]

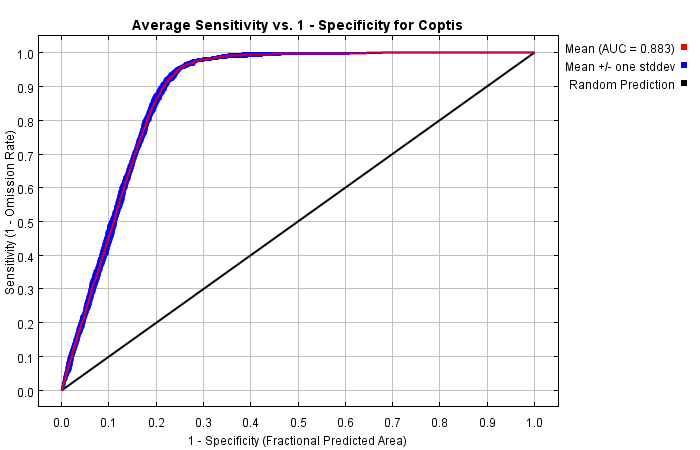

Supplement: Supplementary file 4 [file Image_4.png]

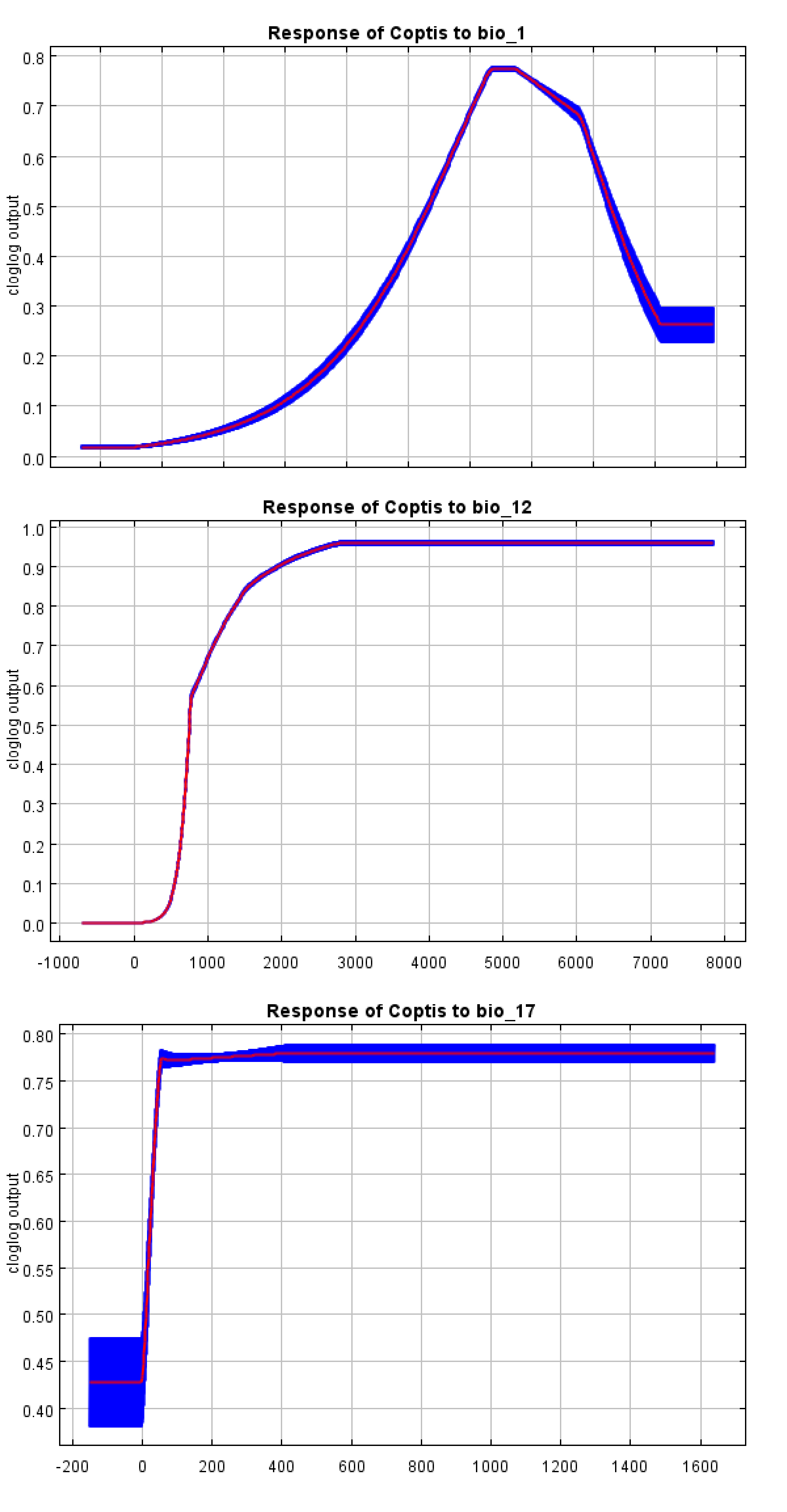

Supplement: Supplementary file 5 [file Image_5.jpeg]

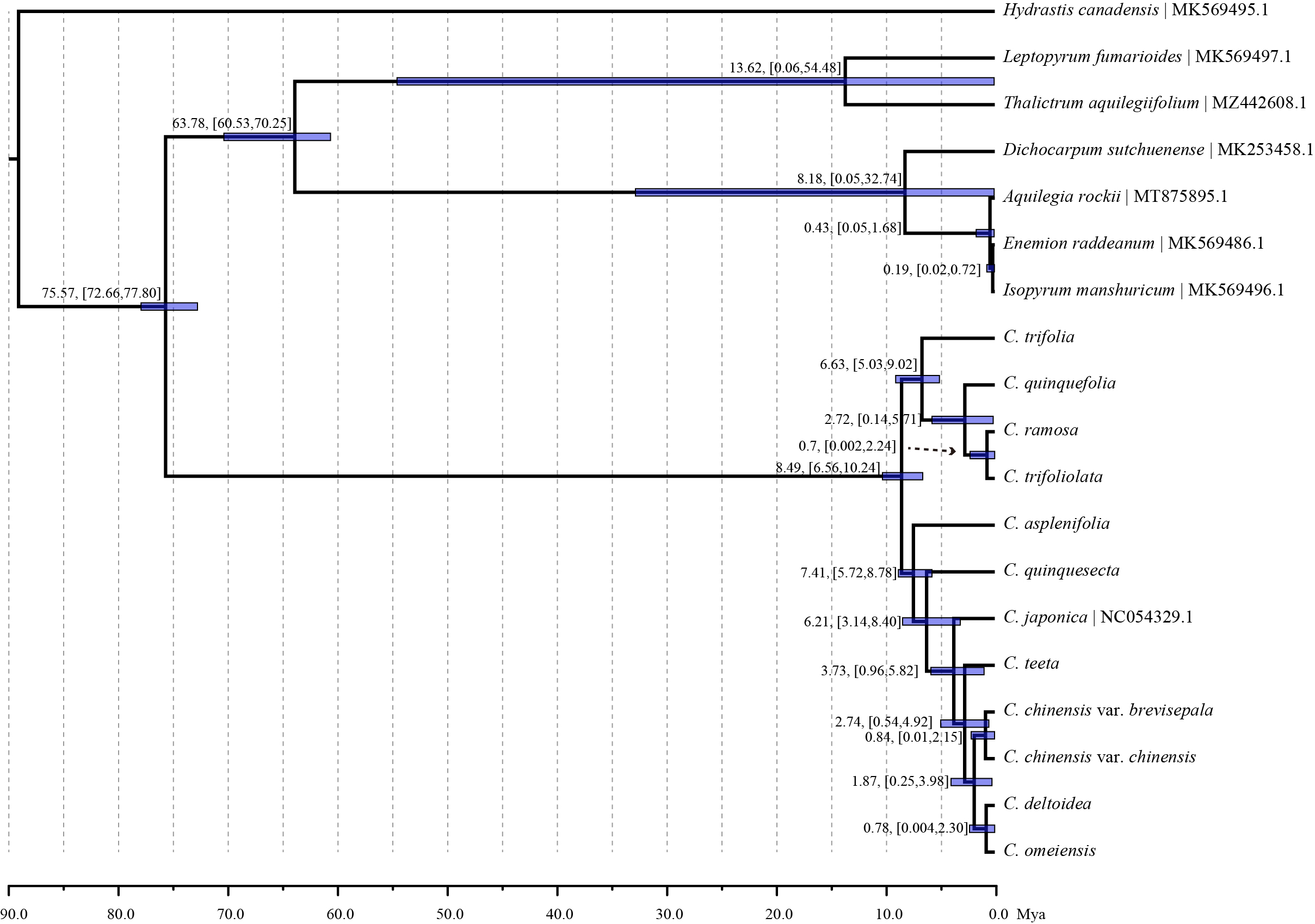

Supplement: Supplementary file 6 [file Image_6.jpeg]

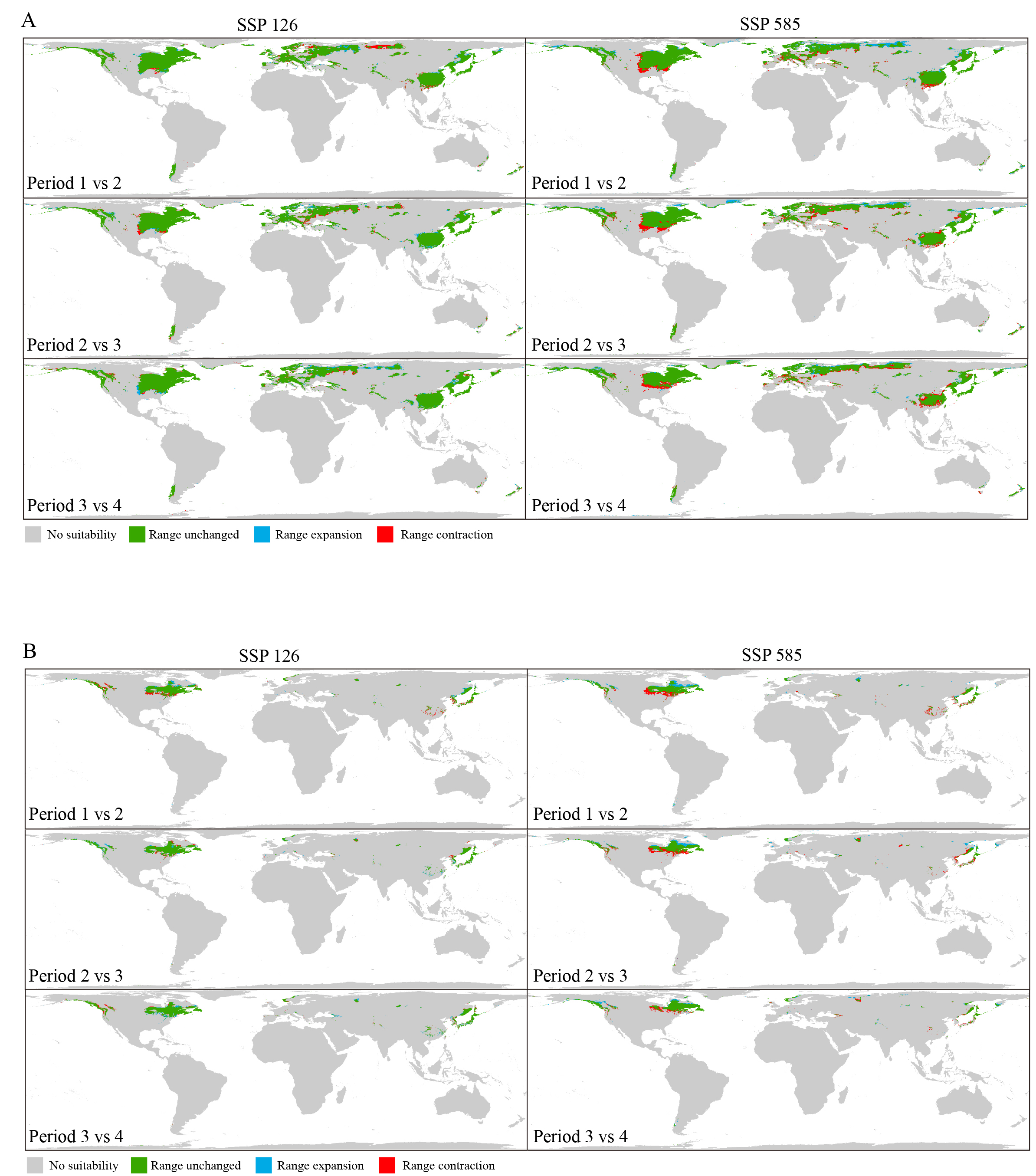

Supplement: Supplementary file 7 [file Image_7.jpeg]
